# Efficacy and Safety of Controlled Ovarian Stimulation With or Without Letrozole Co-administration for Fertility Preservation: A Systematic Review and Meta-Analysis

**DOI:** 10.3389/fonc.2020.574669

**Published:** 2020-10-07

**Authors:** Benedetta Bonardi, Claudia Massarotti, Marco Bruzzone, Oranite Goldrat, Giorgia Mangili, Paola Anserini, Stefano Spinaci, Luca Arecco, Lucia Del Mastro, Marcello Ceppi, Isabelle Demeestere, Matteo Lambertini

**Affiliations:** ^1^Research Laboratory on Human Reproduction, Université Libre de Bruxelles (ULB), Brussels, Belgium; ^2^Obstetrics and Gynecology Unit, IRCCS San Raffaele Scientific Institute, Milan, Italy; ^3^Physiopathology of Human Reproduction Unit, IRCCS Ospedale Policlinico San Martino, Genova, Italy; ^4^Clinical Epidemiology Unit, IRCCS Ospedale Policlinico San Martino, Genova, Italy; ^5^Fertility Clinic, CUB-Hôpital Erasme, Brussels, Belgium; ^6^Division of Breast Surgery, Ospedale Villa Scassi, Genova, Italy; ^7^Department of Internal Medicine and Medical Specialties (DiMI), School of Medicine, University of Genova, Genova, Italy; ^8^Department of Medical Oncology, U.O.C. Clinica di Oncologia Medica, IRCCS Ospedale Policlinico San Martino, Genova, Italy; ^9^Breast Unit, IRCCS Ospedale Policlinico San Martino, Genova, Italy

**Keywords:** fertility, controlled ovarian stimulation, letrozole, gonadotropins, breast cancer

## Abstract

**Background:** The co-administration of letrozole during controlled ovarian stimulation (COS) with gonadotropins is used to limit the potentially harmful effects of a supra-physiological rise in estrogen levels on hormone-sensitive cancers. However, the efficacy and safety of adding letrozole to COS remain debated.

**Methods:** This is a systematic review and meta-analysis of published studies that compared the efficacy and safety of COS with co-administration of letrozole vs. COS without letrozole in all patient populations. A secondary analysis was done including only the studies in breast cancer patients. The primary efficacy endpoint was the number of retrieved mature Metaphase II (MII) oocytes. Secondary efficacy and safety endpoints were total number of oocytes, maturation rate, fertilization rate, number of cryopreserved embryos, peak estradiol levels, progesterone levels, and total gonadotropin dose. Data for each endpoint were reported and analyzed thorough mean ratio (MR) with 95% confidence interval (CI).

**Results:** A total of 11 records were selected including 2,121 patients (990 patients underwent COS with letrozole and 1,131 COS without letrozole). The addition of letrozole to COS did not have any negative effect on the number of mature oocytes collected (MR = 1.00, 95% CI = 0.87–1.16; *P* = 0.967) and the other efficacy endpoints. COS with letrozole was associated with significantly decreased peak estradiol levels (MR = 0.28, 95% CI = 0.24–0.32; *P* < 0.001). Similar results were observed in the secondary analysis including only breast cancer patients.

**Conclusions:** These findings are reassuring on the efficacy and safety of COS with gonadotropins and letrozole and are particularly important for fertility preservation in women with hormone-sensitive cancers.

## Introduction

Over the last years, cancer death rate has been continuously dropping thanks to improvement in screening techniques and therapies ensuring early diagnosis and increased survival (1). Life-saving treatments such as chemotherapy or radiotherapy have several potential long-term adverse effects including gonadotoxicity (2–8). The subsequent risk of treatment-related infertility and the loss of ovarian endocrine function represent important causes of distress for patients who are diagnosed during their reproductive years (9–11). Therefore, scientific societies strongly recommend fertility consultation before initiation of anticancer treatments in all patients of childbearing age (12–15).

In the last decades, oocyte and embryo cryopreservation have become standard procedures for fertility preservation (12–15). In order to increase the chance for success, controlled ovarian stimulation (COS) with high doses of gonadotropins is needed to maximize the number of oocytes retrieved and stored (16). COS exposes women to supra-physiological estrogen levels, raising concerns about the safety of the procedure in patients with hormone-sensitive cancers (17, 18). The use of both letrozole and tamoxifen was proposed, alongside classic COS protocols, to avoid unnecessary and potentially harmful effects of the rise in estrogen levels on the cancer (19, 20). Letrozole is an aromatase inhibitor that blocks androgen conversion into estrogen and it is used “off label” in infertility treatment in many countries, especially as ovulation inductor for women with either anovulatory cycles (21), including those with polycystic ovary syndrome (PCOS) (22), or unexplained infertility before planned intercourses or intra-uterine insemination (IUI) (23). Co-treatment with letrozole was proposed also alongside the COS for *in vitro* fertilization (IVF) in infertile women (24). However, the warning letter published by the original manufacturer still limits its general acceptance. Indeed, the safety concerns related to an increased number of reported malformations in pregnancies resulting from protocols that included letrozole were based only on a single abstract (including 150 babies from 130 pregnancies, compared to a large group of spontaneous low-risk pregnancies) and never confirmed by larger and methodologically sounder studies (25–28). Therefore, the concerns related to potential risks of congenital malformations have been dispelled by the scientific community, but the warning remains.

In terms of efficacy, some studies showed that letrozole co-administration was associated with comparable or even better oocyte yield than traditional protocols, without increasing serum estradiol levels (19, 29, 30), while others have demonstrated a reduction in the number of growing follicles, oocytes retrieved, and pregnancies as well as an increased incidence of cycle cancellations (31, 32). Moreover, data are not homogeneous, with most studies comparing COS with letrozole in oncologic patients to infertile women or donors as controls (29, 33, 34).

Because of the aforementioned controversial data about this important issue, we performed a systematic review and meta-analysis to clarify the efficacy and safety of adding letrozole to COS for IVF.

## Materials and Methods

This was a quantitative synthesis of studies that compared the efficacy and safety of COS with co-administration of letrozole (letrozole cohort) vs. COS without letrozole (no-letrozole cohort).

### Study Endpoints

The primary efficacy endpoint was the number of retrieved mature Metaphase II (MII) oocytes. Secondary efficacy endpoints were total number of retrieved oocytes, maturation rate, and fertilization rate. Other secondary safety endpoints were peak estradiol levels, total gonadotropin dose, and length of the stimulation.

Pregnancy rate, live birth rate, relapse rate, and disease-free survival in cancer patients, adverse events, and progesterone levels were other pre-planned endpoints of interest. However, they could not be analyzed due to lack of data among the included studies.

As secondary analysis, the role of COS with or without letrozole was investigated specifically in the breast cancer patient population. All the analyses were repeated by including only the three studies that included breast cancer patients in both the letrozole and no-letrozole cohorts (35–37).

### Data Sources and Search Strategy

A systematic literature search of PubMed was conducted to identify studies investigating protocols of COS with letrozole compared to those without letrozole. The search was not limited to studies about cancer patients who needed to cryopreserve their oocytes or embryos, but included also infertile patients and COS for elective fertility preservation. The search was restricted to full papers written in English and reporting original data; no restriction in terms of year of publication was applied. The final date of search was March 31, 2020. The terms used for the search strategy were “letrozole,” “aromatase inhibitor,” “controlled ovarian stimulation,” “fertility preservation,” “cancer,” “breast cancer,” “oocyte vitrification,” and “oocyte freezing.” Boolean operators were used to connect specific search keywords.

The effective combination of search terms was designed and organized by one reviewer (BB) and discussed with two other reviewers (ID and ML). The titles and abstracts obtained from the search were analyzed independently by two reviewers (BB and ML), and a third author (ID) evaluated the search results in order to apply the eligibility criteria.

### Article Selection

Records eligible for this analysis had the following features: (a) studies comparing COS with or without letrozole; (b) in the experimental group, letrozole had to be included for the whole COS. Records with the following characteristics were excluded: (a) studies in which letrozole was given only for a few days and not for the whole duration of COS; (b) studies that used letrozole only for ovulation induction; (c) studies written in languages other than English; (d) studies without control group; and (e) studies that compared COS with letrozole vs. COS plus other drugs.

Two investigators (BB and ML) independently extracted data from all the eligible studies. From each eligible record, the following variables were collected: first author, year of publication, sample size and type of COS (letrozole and no letrozole), patients' characteristics (indications, age), characteristics of COS cycle (trigger method, estradiol level at triggering, total gonadotropin dose, and number of days of stimulation), efficacy outcomes (number of mature MII oocytes, total number of oocytes retrieved, number of cryopreserved mature oocytes, maturation rate, fertilization rate, number of cryopreserved embryos, and pregnancy rate/live birth rate), relapse rate and disease-free survival (in cancer patients), adverse events, and progesterone levels when available.

### Statistical Analysis

Mean values with standard deviation or odds ratios (ORs) with 95% confidence intervals (CIs) were collected for all endpoints of interest (number of MII oocytes, total number of collected oocytes, maturation rate, fertilization rate, peak estradiol levels, total gonadotropin dose, and length of the stimulation). Statistical analysis was conducted with a random-effects model.

In order to analyze each endpoint and to compare the performances of COS with or without letrozole, data were studied via mean ratios (MRs), 95% CI, and *P*-values. A MR value >1 indicates that for a specific endpoint, the letrozole cohort has higher values while a MR < 1 means that the study favors standard COS without letrozole.

*P* < 0.05 were considered statistically significant. To evaluate heterogeneity among studies, *I*^2^ values and relative *P*-values were also reported. A sensitivity analysis for each endpoint was performed to assess if the results were mostly driven by one or more studies.

## Results

**Figure 1:**
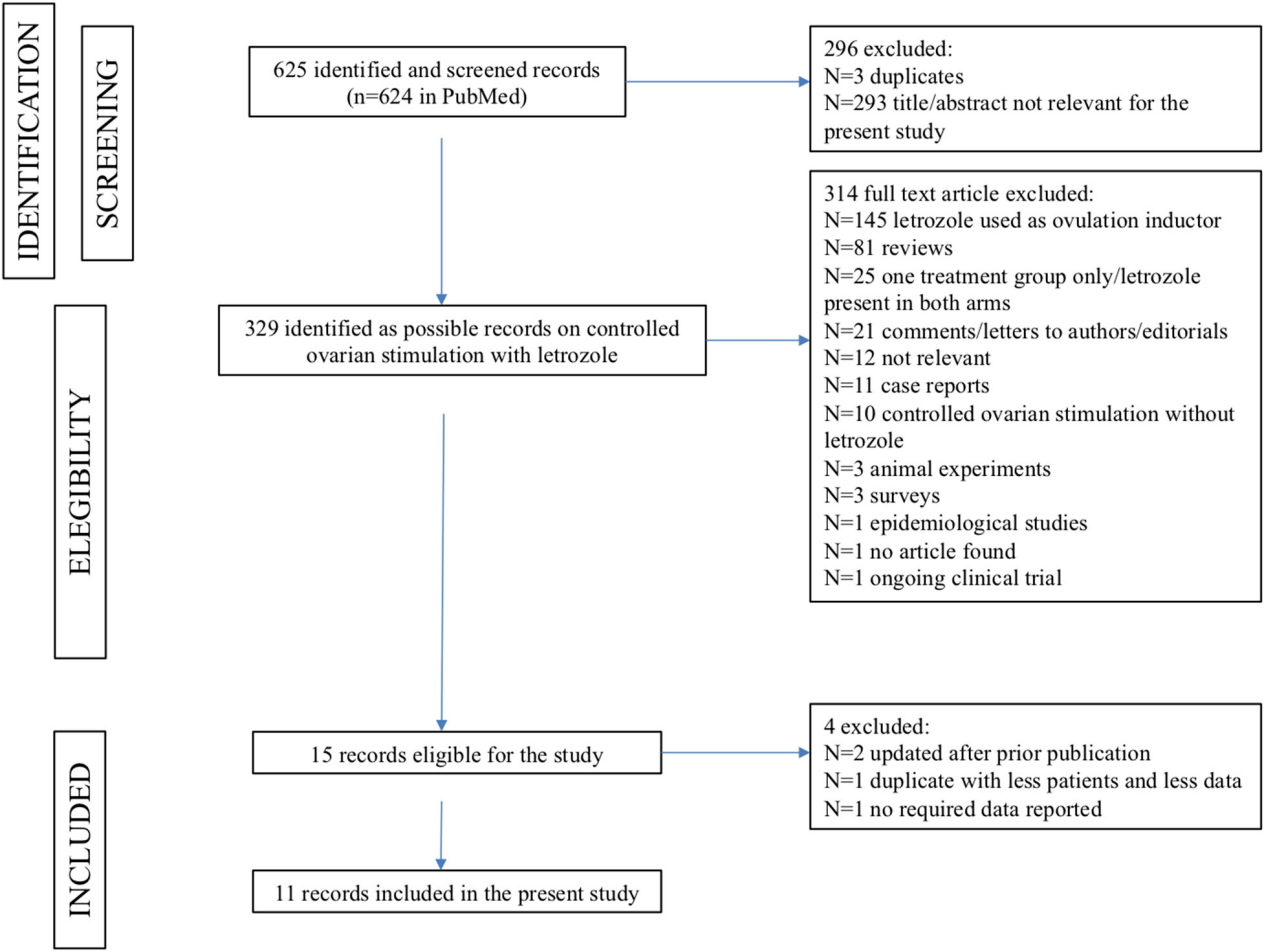
The PRISMA flowchart summarizing the process for identifying the records to include in the present meta-analysis.

The search strategy returned 625 records: after applying the inclusion and exclusion criteria, 15 records were potentially eligible for this meta-analysis (Figure 1). Among them, three records were excluded because they referred to the same study: in two cases [Goldrat et al. (38) vs. Goldrat et al. (34) and Cakman et al. (39) vs. Quinn et al. (36)], the article with the most updated data was selected (34, 36); for the other case [Haas et al. (24) vs. Haas et al. (40)], the least recent paper was included because of a larger sample size and the reporting of endpoints considered in the present meta-analysis (24). One article was excluded because it did not provide the required data for statistical analysis (41).

Therefore, a total of 11 records were selected for the current meta-analysis, including 2,121 patients, of whom 990 underwent COS with letrozole and 1,131 underwent COS without letrozole (24, 29, 33–37, 42–45). Six studies were conducted in cancer patients only (35–37, 42, 43, 45); one study in infertile patients only (24). COS with letrozole in cancer patients was compared to COS without letrozole in infertile controls in two studies (29, 34), to COS without letrozole in healthy elective fertility preservation patients in another study (33), and to both cancer patients and healthy elective fertility preservation patients in another study (44).

The main characteristics of the included studies are summarized in Table 1.

**Table 1:** Main characteristics of the included studies and type of protocol of controlled ovarian stimulation.

		**Number of patients**		**Type of patients**		**Age**		**COS protocol**		
**Author**	**Year**	**Letrozole**	**No letrozole**	**Letrozole**	**No letrozole**	**Letrozole**	**No letrozole**	**Follicular development**	**Ovulation suppression**	**Trigger**
Sonigo et al.	2019	94	83	BC	BC	33.5 ±4.5 mean ± SD	33.6 ± 3.3 mean ± SD	rFSH	GnRH antagonist	GnRH agonist
Goldrat et al.	2019	23	24	BC	IN	30.4 ± 3.8 mean ±SD	30.8 ± 3.9 mean ±SD	rFSH	GnRH antagonist	GnRH agonist (for BC and at risk of OHSS) for the others hCG
Ben Harush et al.	2019	145	273	BC	K+EL	33.7 ± 5.1 mean ± SD	30.0 ± 7.5 mean ± SD	rFSH	GnRH antagonist	GnRH agonist
Haas et al.	2017	87	87	IN	IN	36.5 ± 4.1 mean ± SD	37.0 ± 3.8 mean ± SD	rFSH	GnRH antagonist + rLH or hMG	hCG+GnRH agonist (GnRH agonist only for patients at risk of OHSS)
Quinn et al.	2017	151	40	BC (ER+)	BC (ER-)	NR	NR	rFSH	GnRH antagonist	hCG or GnRH agonist (decision taken singularly depending upon size of the follicular cohort and perceived risk of OHSS)
Pereira et al.	2016	220	439	BC	EL	36 (33-38) median (IQ range)	37 (34–39) median (IQ range)	rFSH	GnRH antagonist	hCG
Johnson et al.	2013	22	28	BC + endometrial k	BC+K	31.2 (19–43) mean (95% CI)	31.2 (21–41) mean (95% CI)	rFSH ± LH support	GnRH antagonist	hCG ± GnRH agonist
Revelli et al.	2013	50	25	BC (ER+)	BC (ER-)	34.4 ± 5.2 mean ± SD	35.1± 4.9 mean ± SD	rFSH or hMG	GnRH antagonist / long GnRH agonist	hCG
Checa Vizcaíno et al.	2012	9	10	BC	K	32 ± 2.87 mean ± SD	28 ± 4.13 mean ± SD	rFSH	GnRH antagonist	GnRH agonist
Domingo et al.	2012	142	66	BC	K	33.2 ± 4.3 mean ± SD	30.6 ± 5.7 mean±SD	rFSH	GnRH antagonist	GnRH agonist
Oktay et al.	2006	47	56	BC	IN	36.4 ± 3.6 mean ± SD	36.9 ± 3.9 mean ± SD	rFSH	GnRH agonist	hCG

*COS, controlled ovarian stimulation; BC, Breast cancer; K, cancer; IN, infertile; EL, Elective; ER -, Estrogen receptor negative; ER +, Estrogen receptor positive; rFSH, recombinant follicle-stimulating hormone; GnRH, gonadotropin-releasing hormone; rLH, recombinant lutenizing hormone; hMG, human menopausal gonadotropin; hCG, human chorionic gonadotropin; BC, breast cancer; OHSS, ovarian hyperstimulation syndrome*.

**Figure 2:**
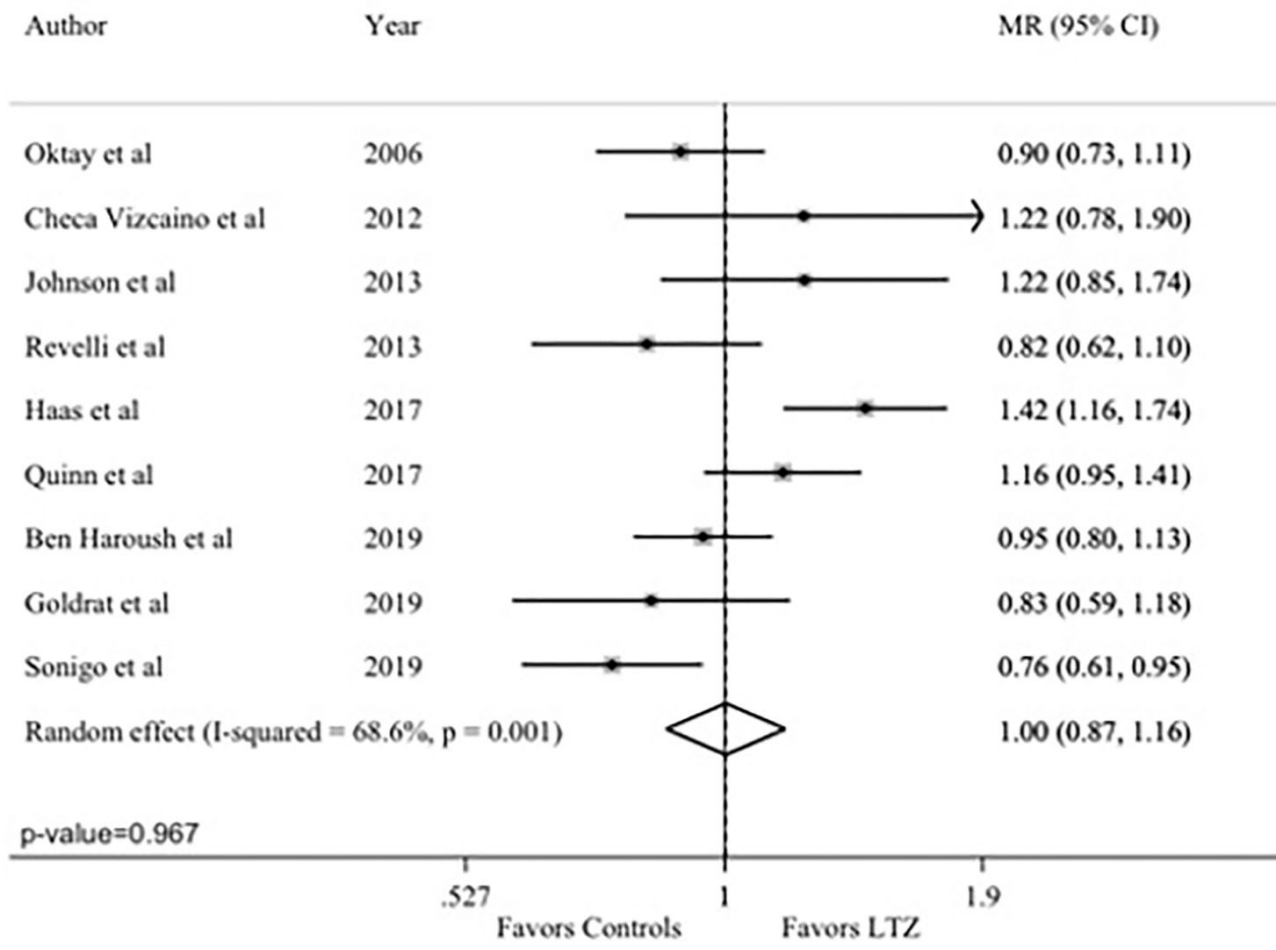
Primary endpoint: number of Metaphase II (MII) oocytes. MR, mean ratios; CI, confidence intervals; LTZ, letrozole.

The total number of MII oocytes, as quantitative marker of efficacy, was reported in nine studies (24, 29, 34–37, 42–44). No difference between the letrozole and no-letrozole cohorts was found with a MR value of 1.00 (95% CI = 0.87–1.16; *P* = 0.967; Figure 2). Heterogeneity was high (*I*^2^ = 68.6%; *P* = 0.001). Sensitivity analysis is reported in Supplementary Table 1.

**Figure 3:**
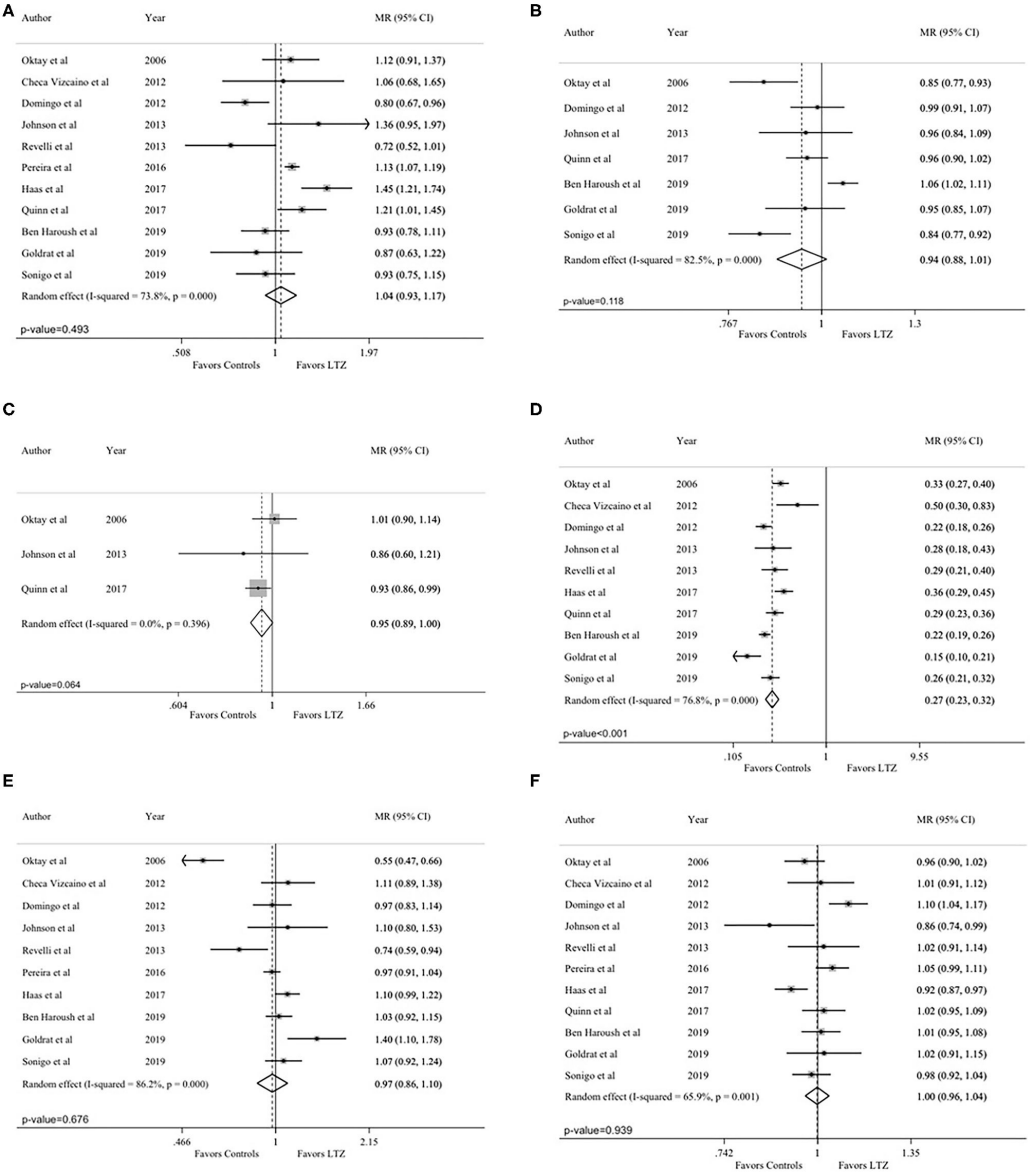
Secondary endpoints: **(A)** total number of retrieved oocytes; **(B)** maturation rate; **(C)** fertilization rate; **(D)** peak estradiol levels; **(E)** total gonadotropin dose; **(F)** length of the stimulation. MR, mean ratios; CI, confidence intervals; LTZ, letrozole.

The total number of retrieved oocytes was reported in all studies (24, 29, 33–37, 42–45). No difference between the letrozole and no-letrozole cohorts was found (MR = 1.04; 95% CI = 0.93–1.17; *P* = 0.493; Figure 3A). Heterogeneity was high (*I*^2^ = 73.8%; *P* < 0.001). Sensitivity analysis is reported in Supplementary Table 2.

Seven studies reported on maturation rate (29, 34, 36, 37, 43–45). Higher maturation rate was observed in the no-letrozole cohort; however, the difference was not statistically significant (MR = 0.94, 95% CI = 0.88–1.01, *P* = 0.118, Figure 3B). Heterogeneity was high (*I*^2^ = 82.5%; *P* < 0.001). Sensitivity analysis is reported in Supplementary Table 3.

Fertilization rate was reported in three studies (29, 36, 43). A higher fertilization rate was observed in the no-letrozole cohort; however, the difference was not statistically significant (MR = 0.95, 95% CI = 0.89–1.00, *P* = 0.064; Figure 3C). No heterogeneity was observed (*I*^2^ = 0.0%; *P* = 0.396). Sensitivity analysis is reported in Supplementary Table 4.

Peak estradiol levels were reported in 10 studies (24, 29, 34–37, 42–45). Estradiol levels were significantly lower in the letrozole cohort (MR = 0.27, 95% CI = 0.23–0.32; *P* < 0.001; Figure 3D). Heterogeneity was high (*I*^2^ = 76.8%; *P* < 0.001). Sensitivity analysis is reported in Supplementary Table 5.

Total gonadotropin dose was reported in 10 studies (24, 29, 33–35, 37, 42–45). No statistically significant difference was observed between the letrozole and no-letrozole cohorts (MR = 0.97, 95% CI = 0.86–1.10, *P* = 0.676; Figure 3E). Heterogeneity was high (*I*^2^ = 86%, *P* < 0.001). Sensitivity analysis is reported in Supplementary Table 6.

Length of the stimulation was reported in all included studies (24, 29, 33–37, 42–45). There was no difference between the letrozole and no-letrozole cohorts (MR =1.00, 95% CI = 0.96–1.04, *P* = 0.939; Figure 3F). Heterogeneity was high (*I*^2^ = 65.9%; *P* = 0.001). Sensitivity analysis is reported in Supplementary Table 7.

**Figure 4:**
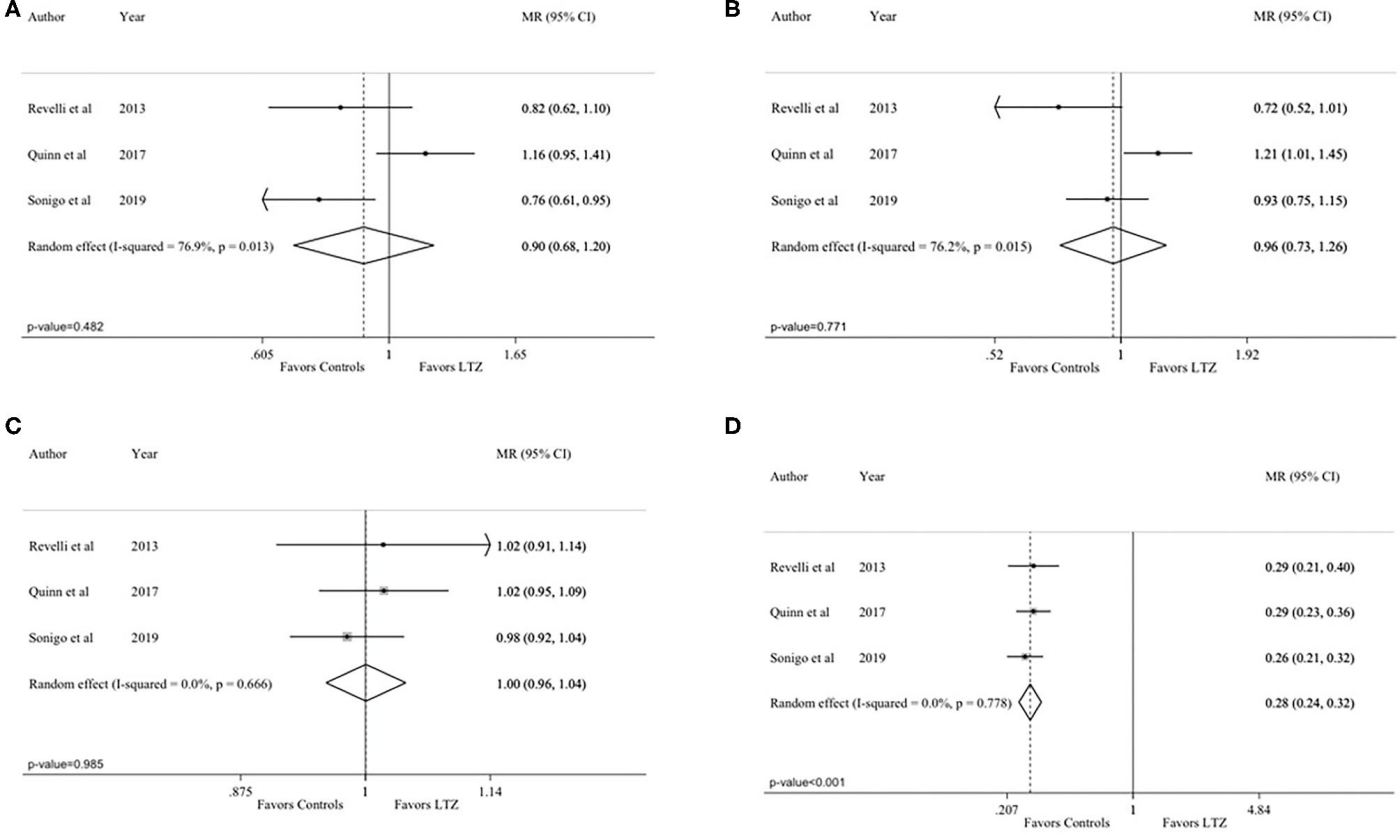
Analysis including only the studies comparing breast cancer patients in both the letrozole and no-letrozole groups: **(A)** number of Metaphase II (MII) oocytes; **(B)** total number of retrieved oocytes; **(C)** length of the stimulation; **(D)** peak estradiol levels. MR, mean ratios; CI, confidence intervals; LTZ, letrozole.

All the analyses were repeated by including only the three articles comparing breast cancer patients in both the letrozole and no-letrozole cohorts (35–37). Based on data availability, four endpoints (number of MII oocytes, total number of oocytes, length of the stimulation, and peak estradiol levels) could be analyzed. The observed results were consistent with those of the primary analysis. No difference between the letrozole and no-letrozole cohorts was observed in terms of total number of MII oocytes (MR = 0.90; 95% CI = 0.68–1.20; *P* = 0.482; Figure 4A; *I*^2^ = 76.9% and *P* = 0.013), total oocytes retrieved (MR = 0.96; 95% CI = 0.73–1.26; *P* = 0.771; Figure 4B; *I*^2^ = 76.2%; *P* = 0.015), and length of the stimulation (MR = 1.00, 95% CI = 0.96–1.04, *P* = 0.985; Figure 4C; *I*^2^ = 0.0%; *P* = 0.666). Peak estradiol levels were significantly lower in the letrozole group as compared to the no-letrozole group (MR = 0.28, 95% CI = 0.24–0.32; *P* < 0.001; Figure 4D; *I*^2^ = 0.0% and *P* = 0.778).

## Discussion

While letrozole as an ovulation inductor is well-known and widely used (21, 22, 46), its role alongside a COS protocol is less studied and therefore less used in infertile patients, due to conflicting results and the safety warning of the producer. However, in the last years, it has become the standard of care for COS in patients with hormone-sensitive cancers to avoid potentially harmful supra-physiological estradiol levels (47), which are the main reason for oncologists to oppose oocyte or embryo cryopreservation (18). However, the evidence on the use of COS protocol that include letrozole is based on few observational studies, most of them with a small sample size and heterogeneous in nature.

The present meta-analysis aimed to assess the efficacy and safety of letrozole co-administration during COS. It showed that the addition of letrozole to COS does not have a negative effect on the number of mature oocytes collected and on other efficacy endpoints, while it is associated with significantly decreased peak estradiol levels which may be of great importance particularly in patients with hormone-sensitive cancers.

A high heterogeneity among studies was observed in the majority of the analysis. This may be due to study design (none was a randomized trial), their low sample size, the different cohorts of patients included also in terms of age, as well as the non-homogeneous COS protocols. For example, Ben Haroush et al. included in the no-letrozole cohort healthy women who elected to have their oocyte cryopreserved for social reasons and very young patients (26.5 ± 7.1 years) with non-hormone-sensitive cancers (44). Both these groups of women are expected to be high-responders, but surprisingly, the authors reported similar number of retrieved oocytes between groups with a slightly higher maturation rate in favor of the breast cancer cohort. The sensitivity analysis reported in Supplementary Table 3 showed that, after excluding the study by Ben Haroush et al., maturation rate results become statistically significant in favor of the no-letrozole cohort, supporting that this study strongly weights on the final statistical results for this parameter.

The study design, and specifically the choice of the controls, is the feature associated with the highest risk of bias for the included studies. By comparing results between cancer patients and healthy infertile women with the latter probably having a worse prognosis at start, a selection bias becomes impossible to avoid, especially in an observational study. Using healthy patients who elected to have their fertility preserved for social reasons is probably a more accurate choice; however, literature is not univocal on ovarian response to COS in cancer patients before gonadotoxic therapies, not excluding a worse ovarian reserve even before starting anticancer therapies (48, 49). Study comparing cancer patients in both study groups usually had smaller sample size and did not exclude potential bias due to the impact of the cancer type. Only three studies included exclusively breast cancer patients in both study cohorts. To specifically investigate the performance of COS with or without letrozole in breast cancer patients, we performed a secondary analysis that showed no influence of letrozole on all the evaluated efficacy endpoints. However, some issues remain to be clarified also in this setting. For example, *BRCA*-mutated women, which are described by some reports as less fertile (50, 51), have more frequently hormone receptor-negative cancers; therefore, they are more likely to be included in the no-letrozole cohorts. Recent data, demonstrating the safety of pregnancy in breast cancer survivors with germline *BRCA* pathogenic variants (52), further highlight the need to pursue with additional research efforts to define the optimal fertility preservation approaches in these patients. Only one study in this meta-analysis compared infertile patients both in the letrozole and no-letrozole cohorts (24). The authors hypothesized a beneficial effect of letrozole on ovarian response because of an androgen-mediated increase of FSH receptors on granulosa cells, as it was seen in primates (53). Their results in terms of number of oocytes and blastocysts obtained are promising, but the study design is retrospective and it analyzed only 174 IVF cycles. A well-designed randomized trial would be better suited to confirm or deny their findings.

Another important potential explanation for the heterogeneity among studies is the ovulation trigger criteria that were used. In an earlier study, Oktay et al. showed lower oocyte maturation rates when trigger was achieved at a leading follicle size of 17 mm (29). Once trigger was done at a follicular size of 19–21 mm, maturation rates improved. In several studies included in our meta-analysis, ovulation trigger was performed in both groups either at 17 mm or “when appropriate” (24, 35, 37, 44). This issue may account for the lower oocyte maturation rate in the letrozole cohort as compared to the non-letrozole cohort.

Importantly, the core outcome of fertility research should be the live birth rate being the ultimate chance that a specific treatment gives a patient the possibility to have a baby. Unfortunately, a meta-analysis on this outcome is not yet possible. Only one study included in our metanalysis reported this outcome in both the letrozole and no-letrozole cohorts (43). In this study, out of 50 patients, only six returned to thaw the embryos, one in the letrozole cohort and five in the no-letrozole cohort. For the patients who received COS with letrozole, one twin pregnancy via gestational carrier was obtained; it was complicated by pre-eclampsia, and two babies were born pre-term with a cesarean section. Among the five patients who received COS without letrozole only, three had their embryos transferred (the embryos did not survive the thawing for the other two). One patient used a gestational carrier, the pregnancy had no complications, and the baby was delivered vaginally at term. The other two patients had singleton pregnancies: one was complicated by pre-term labor but managed to deliver at term vaginally; the other was complicated by a baby large for gestational age and was delivered at term via cesarean section (43). Notably, utilization rate of cryopreserved material in cancer patients is reported to be quite low [around 10–23% for frozen embryos (54–56) and 5% for frozen oocytes (57–59)], considering that these women, also those who need to use their cryopreserved oocytes or embryos to have a pregnancy, have to complete their oncological therapies before. With time, more data on the utilization of such material will become available.

In terms of safety concerns, peak estradiol level was lower in the letrozole cohort. These data indirectly confirm the possible protective mechanism of letrozole for patients who are affected by hormone-sensitive cancers including breast tumors. Breast cancer patients who undergo COS with letrozole before starting chemotherapy does not appear to have higher risk of recurrence than those who do not undergo any fertility preservation procedure (60). The safety of this approach has also been shown for patients undergoing neoadjuvant chemotherapy, although the evidence is more limited in this setting (60, 61). The length of the stimulation is another safety parameter; indeed, this is of particular importance for cancer patients who need to start life-saving oncological treatments as soon as possible. Our study shows that standard protocols for COS with or without letrozole have the same stimulation length. Due to the paucity of information reported in the included articles, safety data remain largely incomplete. More evidence is needed on oncological outcomes (i.e., relapse rate, disease-free survival, adverse events, and delay in chemotherapy start) (62) as well as progesterone levels during COS (38, 63).

In conclusion, letrozole co-administration during COS resulted to be as effective as standard COS but with significantly decreased peak estradiol levels, suggesting its increased safety for patients with hormone-sensitive cancers. Although current data are reassuring, more studies, including randomized controlled trials, are needed to finally prove the efficacy and safety of letrozole co-administration during COS, particularly among cancer patients. Moreover, long-term outcomes in terms of both efficacy and safety should be strongly encouraged to be collected.

## Data Availability Statement

The data extracted from the original publications and supporting the conclusions of this article will be made available by the authors, without undue reservation.

## Author Contributions

ID and ML contributed to the conception and design of the study. BB, ID, and ML performed the literature search, study selection, and data extraction that were then reviewed by all authors. MB and MC performed the statistical analysis. The results were interpreted by BB, CM, ID, and ML The initial manuscript was drafted by BB, CM, ID, and ML. All authors revised the manuscript critically for important intellectual content and approved it.

## Conflict of Interest

LDM acted as consultant for Roche, Novartis, Eisai, Pfeizer, Astrazeneca, Ipsen, Eli Lilly, MSD, Seattle Genetics and Genomic Health outside the submitted work. ID acted as consultant for Roche and received speaker honoraria from Novartis. ML acted as a consultant for Roche and Novartis, and received honoraria from Theramex, Takeda, Roche, Lilly, Pfizer and Novartis outside the submitted work. The remaining authors declare that the research was conducted in the absence of any commercial or financial relationships that could be construed as a potential conflict of interest.

## Supplementary Material

Supplementary Table 1 Sensitivity analysis for the endpoint “mature Metaphase II (MII) oocytes.”

Supplementary Table 2 Sensitivity analysis for the endpoint “total number of retrieved oocytes.”

Supplementary Table 3 Sensitivity analysis for the endpoint “maturation rate.”

Supplementary Table 4 Sensitivity analysis for the endpoint “fertilization rate.”

Supplementary Table 5 Sensitivity analysis for the endpoint “peak estradiol levels.”

Supplementary Table 6 Sensitivity analysis for the endpoint “total gonadotropin dose.”

Supplementary Table 7 Sensitivity analysis for the endpoint “length of the stimulation.”
