# Supplementary material for: Efficacy and Safety of Controlled Ovarian Stimulation With or Without Letrozole Co-administration for Fertility Preservation: A Systematic Review and Meta-Analysis
Source: Front Oncol. 2020 Oct 7;10:574669. doi: 10.3389/fonc.2020.574669 (PMC7575927; doi:10.3389/fonc.2020.574669)
Supplement: Supplementary Table 1 — Sensitivity analysis for the endpoint “mature Metaphase II (MII) oocytes.” [file Data_Sheet_1.docx]

**Supplementary Table 1.** Sensitivity analysis for the endpoint “mature Metaphase II (MII) oocytes”.

| **Study excluded** | **Random effect** | | | **I-squared (%)** | **I-sq. P-value** |
| --- | --- | --- | --- | --- | --- |
|  | **MR** | **95% CI** | **P-value** |  |  |
| Oktay et al | 1.02 | 0.86-1.20 | 0.819 | 70.9 | 0.001 |
| Checa Vizcaino et al | 0.99 | 0.85-1.16 | 0.889 | 71.7 | 0.001 |
| Johnson et al | 0.98 | 0.84-1.15 | 0.848 | 71.2 | 0.001 |
| Revelli et al | 1.03 | 0.88-1.20 | 0.747 | 70.1 | 0.001 |
| Haas et al | 0.95 | 0.84-1.07 | 0.395 | 44.7 | 0.081 |
| Quinn et al | 0.98 | 0.83-1.16 | 0.830 | 69.8 | 0.002 |
| Ben Haroush et al | 1.01 | 0.85-1.21 | 0.893 | 71.9 | 0.001 |
| Goldrat et al | 1.02 | 0.87-1.20 | 0.791 | 71.1 | 0.001 |
| Sonigo et al | 1.04 | 0.90-1.21 | 0.561 | 61.9 | 0.010 |

**Supplementary Table 2.** Sensitivity analysis for the endpoint “total number of retrieved oocytes”.

| **Study excluded** | **Random effect** | | | **I-squared (%)** | **I-sq. P-value** |
| --- | --- | --- | --- | --- | --- |
|  | **MR** | **95% CI** | **P-value** |  |  |
| Oktay et al | 1.03 | 0.91-1.17 | 0.629 | 76.4 | <0.001 |
| Checa Vizcaino et al | 1.04 | 0.92-1.17 | 0.525 | 76.4 | <0.001 |
| Domingo et al | 1.08 | 0.97-1.20 | 0.167 | 64.9 | 0.002 |
| Johnson et al | 1.02 | 0.91-1.15 | 0.704 | 75.5 | <0.001 |
| Revelli et al | 1.07 | 0.95-1.19 | 0.250 | 72.0 | <0.001 |
| Pereira et al | 1.02 | 0.89-1.18 | 0.738 | 73.9 | <0.001 |
| Haas et al | 1.00 | 0.90-1.12 | 0.972 | 68.6 | 0.001 |
| Quinn et al | 1.02 | 0.90-1.16 | 0.748 | 75.6 | <0.001 |
| Ben Haroush et al | 1.05 | 0.93-1.19 | 0.397 | 74.0 | <0.001 |
| Goldrat et al | 1.05 | 0.94-1.18 | 0.388 | 75.3 | <0.001 |
| Sonigo et al | 1.05 | 0.93-1.19 | 0.411 | 75.0 | <0.001 |

**Supplementary Table 3.** Sensitivity analysis for the endpoint “maturation rate”.

| **Study excluded** | **Random effect** | | | **I-squared (%)** | **I-sq. P-value** |
| --- | --- | --- | --- | --- | --- |
|  | **MR** | **95% CI** | **P-value** |  |  |
| Oktay et al | 0.96 | 0.90-1.03 | 0.281 | 80.1 | <0.001 |
| Domingo et al | 0.94 | 0.86-1.02 | 0.131 | 85.4 | <0.001 |
| Johnson et al | 0.94 | 0.87-1.02 | 0.144 | 85.4 | <0.001 |
| Quinn et al | 0.94 | 0.86-1.03 | 0.181 | 85.2 | <0.001 |
| Ben Haroush et al | 0.92 | 0.87-0.98 | 0.007 | 59.8 | 0.029 |
| Goldrat et al | 0.94 | 0.87-1.02 | 0.151 | 85.3 | <0.001 |
| Sonigo et al | 0.97 | 0.90-1.03 | 0.300 | 76.6 | 0.001 |

**Supplementary Table 4.** Sensitivity analysis for the endpoint “fertilization rate”.

| **Study excluded** | **Random effect** | | | **I-squared (%)** | **I-sq. P-value** |
| --- | --- | --- | --- | --- | --- |
|  | **MR** | **95% CI** | **P-value** |  |  |
| Oktay et al | 0.92 | 0.86-0.99 | 0.025 | 0.0 | 0.664 |
| Johnson et al | 0.95 | 0.88-1.04 | 0.271 | 35.0 | 0.215 |
| Quinn et al | 0.99 | 0.89-1.11 | 0.924 | 0.0 | 0.375 |

**Supplementary Table 5.** Sensitivity analysis for the endpoint “peak estradiol levels”.

| **Study excluded** | **Random effect** | | | **I-squared (%)** | **I-sq. P-value** |
| --- | --- | --- | --- | --- | --- |
|  | **MR** | **95% CI** | **P-value** |  |  |
| Oktay et al | 0.26 | 0.22-0.31 | <0.001 | 75.7 | <0.001 |
| Checa Vizcaino et al | 0.26 | 0.22-0.30 | <0.001 | 75.5 | <0.001 |
| Domingo et al | 0.28 | 0.23-0.33 | <0.001 | 77.0 | <0.001 |
| Johnson et al | 0.27 | 0.23-0.32 | <0.001 | 79.3 | <0.001 |
| Revelli et al | 0.27 | 0.22-0.32 | <0.001 | 79.2 | <0.001 |
| Haas et al | 0.26 | 0.22-0.30 | <0.001 | 73.6 | <0.001 |
| Quinn et al | 0.27 | 0.22-0.32 | <0.001 | 78.9 | <0.001 |
| Ben Haroush et al | 0.28 | 0.23-0.33 | <0.001 | 75.7 | <0.001 |
| Goldrat et al | 0.28 | 0.24-0.33 | <0.001 | 70.5 | 0.001 |
| Sonigo et al | 0.27 | 0.23-0.32 | <0.001 | 79.4 | <0.001 |

**Supplementary Table 6.**  Sensitivity analysis for the endpoint “total gonadotropin dose”.

| **Study excluded** | **Random effect** | | | **I-squared (%)** | **I-sq. P-value** |
| --- | --- | --- | --- | --- | --- |
|  | **MR** | **95% CI** | **P-value** |  |  |
| Oktay et al | 1.03 | 0.96-1.12 | 0.398 | 58.3 | 0.014 |
| Checa Vizcaino et al | 0.96 | 0.84-1.10 | 0.564 | 87.5 | <0.001 |
| Domingo et al | 0.97 | 0.85-1.12 | 0.710 | 87.7 | <0.001 |
| Johnson et al | 0.96 | 0.85-1.10 | 0.593 | 87.7 | <0.001 |
| Revelli et al | 1.00 | 0.88-1.14 | 0.988 | 86.6 | <0.001 |
| Pereira et al | 0.97 | 0.83-1.14 | 0.754 | 87.7 | <0.001 |
| Haas et al | 0.96 | 0.83-1.10 | 0.560 | 86.6 | <0.001 |
| Ben Haroush et al | 0.97 | 0.84-1.12 | 0.656 | 87.6 | <0.001 |
| Goldrat et al | 0.94 | 0.83-1.07 | 0.344 | 85.9 | <0.001 |
| Sonigo et al | 0.96 | 0.84-1.11 | 0.597 | 87.5 | <0.001 |

**Supplementary Table 7.**  Sensitivity analysis for the endpoint “length of the stimulation”.

| **Study excluded** | **Random effect** | | | **I-squared (%)** | **I-sq. P-value** |
| --- | --- | --- | --- | --- | --- |
|  | **MR** | **95% CI** | **P-value** |  |  |
| Oktay et al | 1.00 | 0.96-1.05 | 0.887 | 67.3 | 0.001 |
| Checa Vizcaino et al | 1.00 | 0.96-1.04 | 0.904 | 69.3 | 0.001 |
| Domingo et al | 0.99 | 0.95-1.02 | 0.456 | 51.4 | 0.030 |
| Johnson et al | 1.01 | 0.97-1.04 | 0.753 | 63.8 | 0.003 |
| Revelli et al | 1.00 | 0.96-1.04 | 0.882 | 69.2 | 0.001 |
| Pereira et al | 0.99 | 0.95-1.03 | 0.708 | 65.0 | 0.002 |
| Haas et al | 1.01 | 0.98-1.05 | 0.518 | 49.2 | 0.039 |
| Quinn et al | 1.00 | 0.95-1.04 | 0.849 | 68.9 | 0.001 |
| Ben Haroush et al | 1.00 | 0.95-1.04 | 0.881 | 69.2 | 0.001 |
| Goldrat et al | 1.00 | 0.96-1.04 | 0.881 | 69.2 | 0.001 |
| Sonigo et al | 1.00 | 0.96-1.04 | 0.987 | 68.8 | 0.001 |
